# Case report: Strategies for improving outcomes in patients with primary ovarian small-cell neuroendocrine carcinoma

**DOI:** 10.3389/fonc.2022.954289

**Published:** 2022-09-23

**Authors:** YingYing Li, Yueling Wu, Ying Zhang, Xiaofang Li

**Affiliations:** ^1^ Department of Obstetrics and Gynecology, Affiliated Hospital of Guangdong Medical University, Zhanjiang, China; ^2^ Graduate School of Guangdong Medical University, Zhanjiang, China; ^3^ Department of Pathology, Affiliated Hospital of Guangdong Medical University, Zhanjiang, China

**Keywords:** ovarian cancer, small cell neuroendocrine tumor, case report, metastatic disease, pulmonary type

## Abstract

Small-cell neuroendocrine carcinoma (SCNEC) of the ovary is a gynecological malignancy characterized by rapid progression and poor prognosis. SCNEC is divided into primary and metastatic tumor. Primary ovarian neuroendocrine cancer is extremely rare and has a low 5-year survival rate. This paper reports the clinical manifestations of a 58-year-old patient with primary ovarian Small-cell neuroendocrine carcinoma and the prognosis after surgical adjuvant chemotherapy. The prevailing literature on this carcinoma is also reviewed and summarized. Our analysis reveals that histopathological examination is the standard diagnostic tool for ovarian SCNEC. We also highlight the importance of comprehensive imaging evaluation, early pathological diagnosis and comprehensive aggressive treatment to the prognosis of patients.

## Background

Neuroendocrine tumors are a group of heterogeneous tumors that originate from different neuroendocrine organs or stem cells. These tumors produce bioactive amines and polypeptide hormones. They usually occur in gastrointestinal pancreas, cervix or ovary (1). Gynecological neuroendocrine tumors are rare and there are no clear guidelines for their clinical management. For instance, ovarian neuroendocrine tumors account for about 2% of all gynecological tumors. Six percent of women have neuroendocrine cells in the ovarian stroma, and these cells contribute to the development primary ovarian neuroendocrine tumors. To date, the origin of neuroendocrine tumors is not clear. These tumors are divided into carcinoid, small cell neuroendocrine tumor, and large cell neuroendocrine tumor (2). Here, we report a patient with primary ovarian Small-cell neuroendocrine carcinoma with brain metastasis. The clinical manifestation, diagnosis, and treatment of the patient are discussed. The patient gave written informed consent to the use of her clinical data for research. The study was approved by the Ethics Committee of the affiliated Hospital of Guangdong Medical University.

## Case presentation

### Basic information

**Figure 1:**
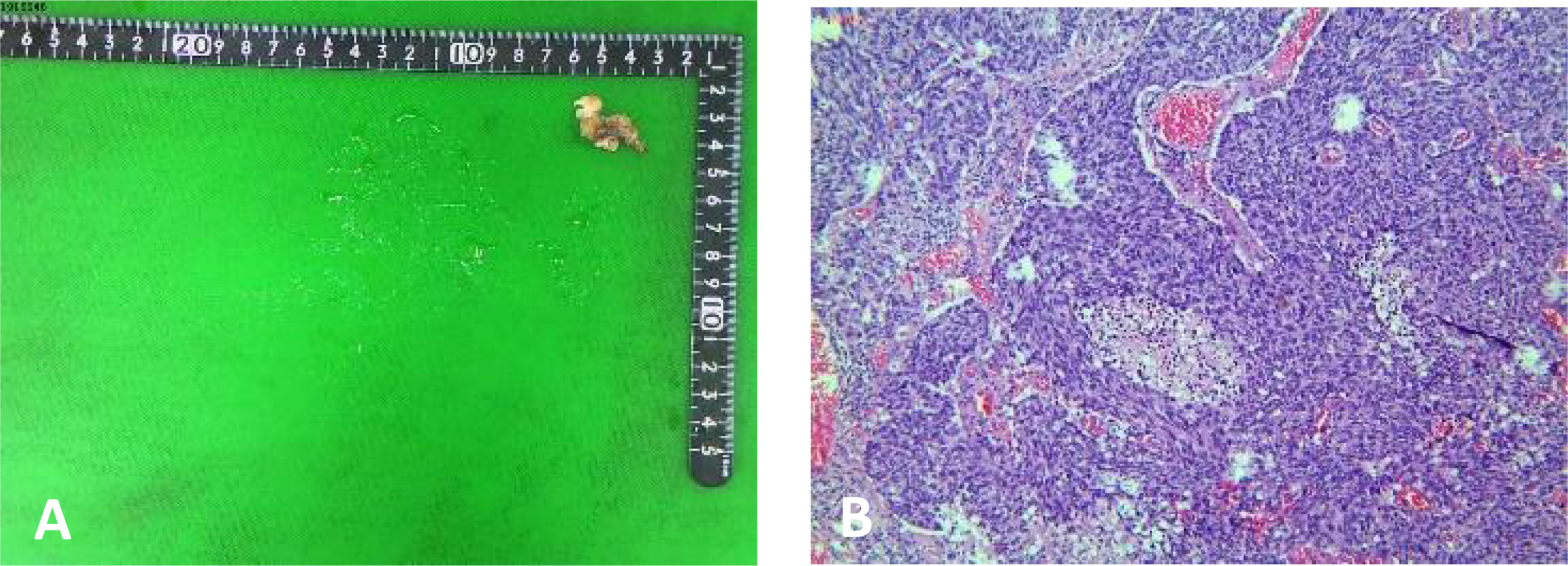
Pathology of brain surgery **(A)** A soft and grey brain solid mass tissue, 2.5 cm * 1.0 cm * 0.5 cm. **(B)** postoperative pathology:left frontal metastatic neuroendocrine carcinoma.

**Figure 2:**
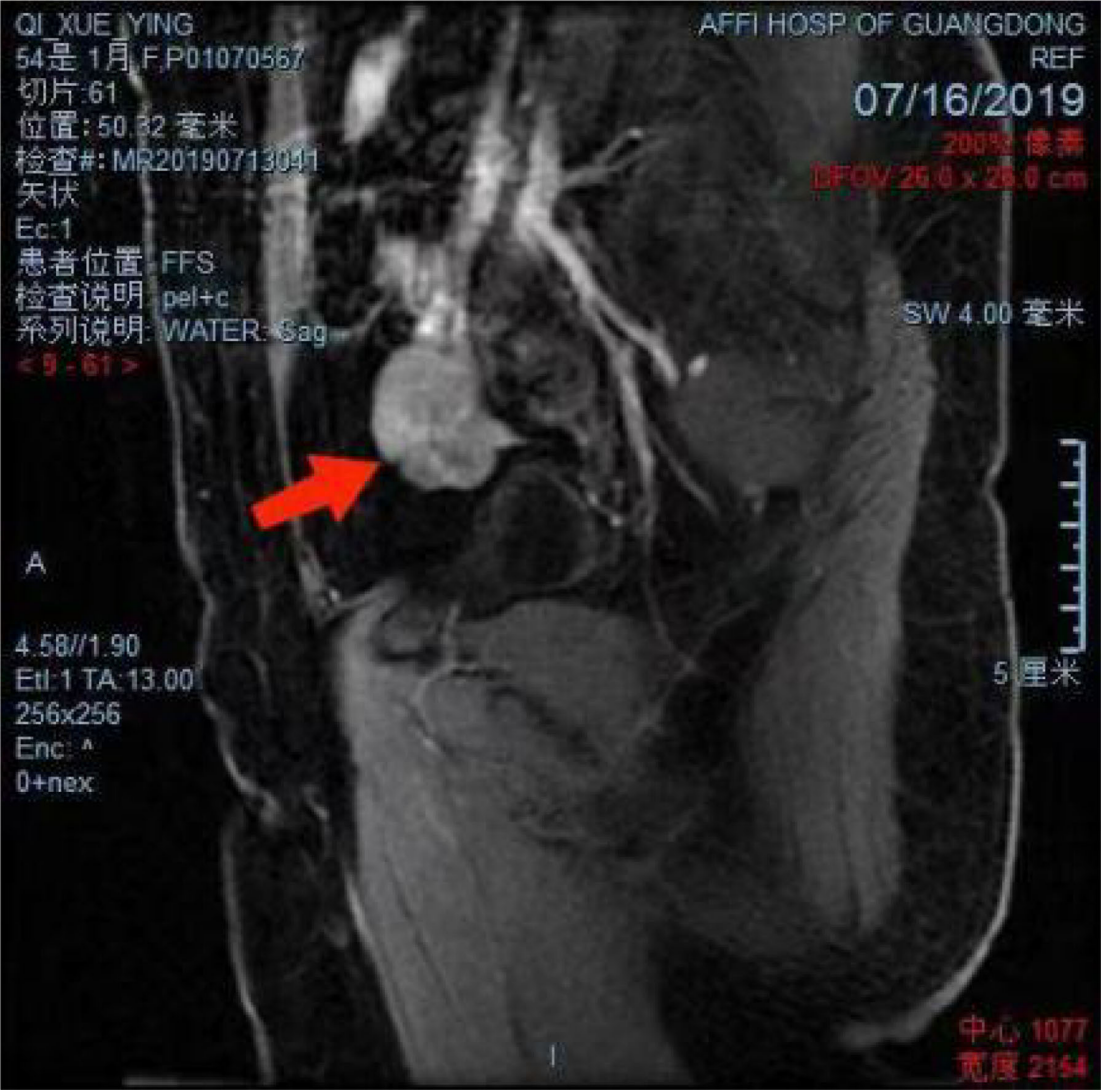
Pelvic magnetic resonance images showing a solid mass of about 2.4 cm * 3.5cm * 3.4cm on the right side of the pelvic region (arrow).

A 58-year-old female patient who underwent brain surgery was suspected to have left frontal metastatic neuroendocrine carcinoma through postoperative pathology (**Figure 1**). After surgery, a pelvic MRI examination revealed a solid mass of about 2.4 cm * 3.5 cm * 3.4 cm on the right side of the pelvic region (**Figure 2**).

### Auxiliary examinations

Gynecologic ultrasound showed irregular hypoecho mass (measuring 4.0 cm * 2. 7 cm) adjacent to the internal iliac arteriovenous vein in the right lower pelvis. Pelvic CTA demonstrated right pelvic cystic-solid mass; the lesion was supplied by blood from the right ovarian artery. In addition, pelvic MRI (**Figure 2**) showed a pelvic right solid mass, measuring about 2.4cm * 3.5cm * 3.4cm, with a clear boundary, local like linear signal connected to the uterus and potential right appendage source of malignant tumor lesions. Tests for serum tumor markers were negative.

### Treatment

The patients received laparoscopic total uterine double attachment resection, bilateral ovarian arteriovenous high ligation, abdominal catheterization and postoperative adjuvant chemotherapy (chemotherapy regimen is shown in **Table 1**).

**Table 1:** Postoperative chemotherapy.

Period	Drug and dose	Side effect
1	Eetoposide 185mg + cisplatin 60mg	III leukocytes、 II thrombocytopenia
2-5	Binding albumin paclitaxel 300mg + cisplatin 55mg	III leukopenia 、 numbness of the hands and feet
6	Illinotecan 200mg + apatinib	III leukopenia
7-8	Carrelizumab 200mg + apatinib	/

### Postoperative pathological examination

**Figure 3:**
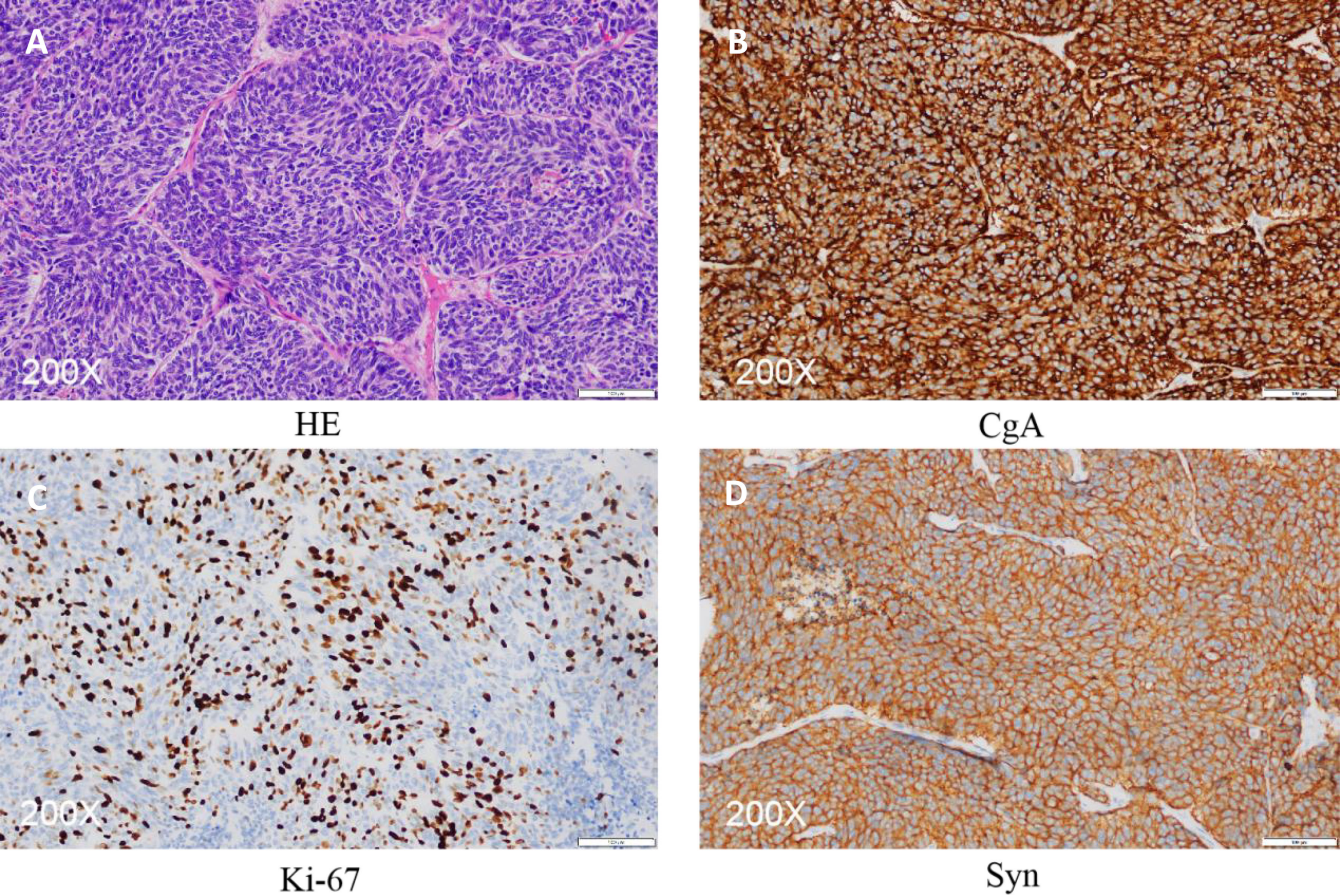
Pathology of ovarian surgery **(A)** HE stain. **(B–D)** Immunohistochemistry images showing the expression of CgA (+), ki-67 index about (30%-40%) and Syn (+).

Postoperative routine pathology showed an intact bilateral ovarian neuroendocrine carcinoma that was confined to the ovary (**Figure 3**). Immunohistochemistry analyses showed CD56 (+), CgA (+) Ki-67 index of about (30% -40%), PAX-8 (-), Syn (+), Vimentin (-) and WT-1 (-).

### Prognosis and follow-up

The patient was still alive at a follow-up performed two years after surgery. There were no signs of recurrence during the follow-up period. However, the patient showed progressive memory decline, recent memory loss, loss of consciousness, behavioral regression, intermittent convulsions which could stop by themselves, and typical brain symptoms.

## Discussion

Primary ovarian small cell carcinoma is a highly malignant gynecological tumor characterized by rapid progression and recurrence. Moreover, it has a low 5-year survival rate, with the 1- and 5- year survival rate being 50% and 10%, respectively (3). According to WHO 2017 Tumors of Female Genital Classification, small cell carcinoma of ovary are classified into Ovary-hypercalcemic Type (SCCOHT) and the Ovary-pulmonary Type (SCCOPT). SCCOPT was first reported by Eichhorn et al. (4) in 1992 (11 cases). However, According to the new WHO classification of Female Genital tumors (5), The concept of SCCOPT no longer exists. But the nomenclature concerning this clinical disorder has been ambiguous and highly depended on histological features. We believe that this case is more consistent with SCCOPT in the old classification, Therefore, we continue to use the old term SCCOPT. In fact, less than 20 cases have been reported on SCCOPT in the past five years. We reviewed and analyzed previous data on ovarian small cell carcinoma as shown in **Table 2**.

**Table 2:** Summary of published small cell carcinoma of the ovary.

Study	Age	n	Type	FIGO Stage	Outcome (follow-up period)
Syed A. Mannan et al.	21	1	SCCOPT(Left) and MOC	IC	DOD(10 months)
Eric M. Sieloff et al.	53	1	SCCOPT (Right)	Unstage	N/A
Parikshaa Gupta et al.	median 20	4	3 SCCOPT (bilateral) 1 SCCOPT (Right) and UEA	IV	1 N/A 1 NED (12 months) 2 AWD (12 months and 30 months)
Parikshaa Gupta et al.	44	1	SCCOPT(Right)	Unstage	N/A
D.Tsolakidis et al.	55	1	SCCOPT (Left)	IIIC	NED(21 months)
Lei Yin et al.	46	1	SCCOPT (bilateral) and UEA	Unstage	DOC(7 months)
Lin LI et al.	median 53	3	2 SCCOPT (Right) 1 SCCOPT (bilateral)	IC/IIIC/IV	2 NED(7 months and 30 months) 1 DOD(12 months)

UEA, Uterine Endometrioid Adenocarcinoma; MOC, Mucinous Ovarian Cancer; SCCOPT, SCCO of pulmonary type; AWD, alive with disease; DOD, dead of disease; DOC, dead of other courses; N/A, not available; NED, noevidence of disease.

Analysis of data on all the cases of lung-type small cell carcinoma generated in the past 5 years showed that all patients were similar in terms of age of onset, clinical symptoms, blood calcium level, pathological characteristics, and prognosis after treatment. However, in 75% of patients with lung small cell carcinoma, bilateral ovaries are involved and are often complicated with other gynecological tumors (5). However, the patient in our study had unilateral ovary carcinoma, with no other gynecological tumors. The patient was a postmenopausal 58-year-old woman who was diagnosed with a pelvic mass without any symptoms after brain surgery. The diagnosis of this condition is based on pathological examination. Previous data have shown that, histopathologically, most SCCOPT cases exhibit an inconspicuous sheet structural pattern, with monomorphic tumor cells showing spotted chromatin, inconspicuous or absent nucleoli, and reduced cytoplasm with nuclear atypia. The tumor cells may be arranged into rosettes in a few cases of SCCOPT. Consistently, our study demonstrates that the tumor cells had a uniform size, nested arrangement, slightly less cytoplasm, high ratio nucleoplasma, less obvious nucleoli, deep-stained cells and follicular space, which is different from features of the high calcium type small cell carcinoma (6).

Immunohistochemical characteristics of ovarian neuroendocrine tumors vary according to the type of tumor. Tumor cells in SCCOPT were cytoplasmically positive for neuroendocrine markers such as chromogranin, neuron-specific enolase, synaptophin and CD56 (membranous). Although chromogranin is the most specific marker, it has low sensitivity. For small cell neuroendocrine carcinoma, it has a positivity rate of about 50%. On the other hand, CD56 has been shown to be the most sensitive marker of neuroendocrine differentiation (62%), followed by CGA (39%) and SYN (26%) (7). These markers show positive results for epithelial cell membrane antigens and may be focal positive for vimentin and cytokeratin. In the present case, CD56 (+), CgA (+), Ki-67 index (30% - 40%), and Syn (+) results were consistent with those reported in previous studies and all of them predict good prognosis.

Clinically, SCCOPT must be distinguished from metastasis of lung and thymic small cell carcinoma to the ovary (8). The two are morphologically indistinguishable, with similar staining results of immune marker. They have neuroendocrine characteristics and can express specific neuroendocrine markers, such as chromogranin and chromopin A. Currently, differential diagnosis of SCCOPT is mainly based on pulmonary primary lesions and clinical history. In a previous study, cytokeratin 20 immunohistochemical findings showed that SCCOPT are perinuclear positive, while lung metastasis cancer show negative CK20 staining, which may provide a useful basis for the authentication and discrimination between SCCOPT and lung metastasis cancer (9). Although immunohistochemical tests did not show whether CK20 was positive, the patient had no history of smoking, chest discomfort or obvious abnormalities in chest CT. Therefore, lung small cell carcinoma was ruled out. Notably, the role of CK20 (+) in distinguishing ovarian primary SCCOPT from lung metastatic small cell carcinoma remains controversial.

Ovarian small cell carcinoma is mainly treated with surgical adjuvant chemotherapy. However, there is no standard chemotherapy regimen. Some studies have proposed that patient sensitivity to platinum drugs is dependent on disease stage, and these drugs may benefit patients with the early stage of the disease. However, of the currently used treatments are not effective. In 2020, Parikshaa Gupta et al. (6) analyzed three patients with stage IV lung small cell cancer who underwent tumor reduction surgery. Two patients received 6 cycles of TC regimen chemotherapy, and one experienced relapse after 1 month and no recurrence was reported after 30 months. The remaining one patient received six cycles of cisplatin plus etoposide chemotherapy plus 2 vaginal brachytherapy after 12 months of follow-up. In 2021, Syed A. Mannan et al (10) performed tumor reduction plus 1 cycle of cisplatin + etoposide chemotherapy in one stage IC patient, who died after 10 months of follow-up. These data demonstrate that a combination of multiagent chemotherapy regimen and paclitaxel can prolong the survival time of patients in the stage of the disease or who develop postoperative recurrence.

In this case, the patient received surgical adjuvant chemotherapy and postoperative chemotherapy regimen (**Table 1**). Irinotecan specifically inhibits DNA topoisomerase I and interferes with DNA replication and cell division, which results in cytotoxicity. In comparison, apatinib is an orally targeted small-molecule anti-angiogenesis drug whereas carilizumab is a humanized anti-PD-1 monoclonal antibody with high affinity.

Chunyan Lan et al. (11) used a low dose of apatinib and carizumab in 10 patients with advanced Cervical Cancer, and obtained an objective remission rate of 55.6% (95% CI, 40.0% to 70.4%), as well as the median progression-free survival was 8.8 months (95% CI, 5.6 months to not estimable). Since the patient in our case had granulocytopia and other symptoms during the first 4 cycles of chemotherapy, it was replaced with second-line therapy. Follow-up data showed that the patient survived for more than 2 years after surgery but developed typical brain symptoms. In conclusion, the use of surgical adjuvant chemotherapy in this case prolonged the survival time of patients. Besides, platinum drugs were also effective for patients with small cell cancer, and their sensitivity was dependent on the disease stage. In summary, patients with small cell carcinoma of ovarian lung type require comprehensive treatment approach regardless of the stage.

## Conclusion

In conclusion, SCCOPT is extremely rare. This cancer is common in postmenopausal women and has no specific clinical manifestations. The pathological and immunological examination of tumor tissues are the main diagnostic tools for this cancer. Positive expression of neuroendocrine markers; CD56, CgA, Syn, and negative results of Vimentin can confirm the diagnosis of SCCOPT. There is no effective treatment for patients with degree of malignancy and risk of relapse. The combination of surgical adjuvant multi-drug chemotherapy and platinum drugs can improve the prognosis of patients. Therefore, comprehensive imaging examination, early pathological diagnosis, and comprehensive treatment are important strategies to improve patient outcomes.

## Data availability statement

The original contributions presented in the study are included in the article/supplementary material. Further inquiries can be directed to the corresponding author.

## Ethics statement

The studies involving human participants were reviewed and approved by the Hospital Ethics Committee of the Affiliated Hospital of Guangdong Medical University. The patients/participants provided their written informed consent to participate in this study. Written informed consent was obtained from the individual(s) for the publication of any potentially identifiable images or data included in this article.

## Author contributions

YL wrote the manuscript. YW contributed substantial advice help to polish the language. YZ conducted the project and revised the whole manuscript. XL was involved in preparing the pathological pictures. All authors contributed to the article and approved the submitted version.

## Funding

This work was supported by the Natural Science Foundation of Guangdong Province (2017A030313559).

## Conflict of interest

The authors declare that the research was conducted in the absence of any commercial or financial relationships that could be construed as a potential conflict of interest.

## Publisher’s note

All claims expressed in this article are solely those of the authors and do not necessarily represent those of their affiliated organizations, or those of the publisher, the editors and the reviewers. Any product that may be evaluated in this article, or claim that may be made by its manufacturer, is not guaranteed or endorsed by the publisher.
